# Piezo1 Activation Prevents Spheroid Formation by Malignant Melanoma SK-MEL-2 Cells

**DOI:** 10.3390/ijms242115703

**Published:** 2023-10-28

**Authors:** Valeria Y. Vasileva, Zuleikha M. Khairullina, Vladislav I. Chubinskiy-Nadezhdin

**Affiliations:** Institute of Cytology, Russian Academy of Sciences, Tikhoretsky Ave. 4, 194064 St. Petersburg, Russia; vvasileva@incras.ru (V.Y.V.);

**Keywords:** Piezo1, aggressive melanoma, Yoda1, cell spheroids

## Abstract

Melanoma is a highly aggressive type of skin cancer produced through the malignant transformation of melanocytes, and it is usually associated with a poor prognosis. Clinically, melanoma has several stages associated with migration and invasion of the cells through the skin’s layers, the rapid spreading of cells and the formation of tumors in multiple organs. The main problem is the emergence of resistance in melanoma to the applied methods of treatment; thus, it is of primary importance to find more crucial signaling pathways that control the progression of this type of cancer and could be targeted to prevent melanoma spreading. Here, we uncover novel aspects of the role of the mechanosensitive ion channel Piezo1 in melanoma tumor formation. Using a combinative approach, we showed the functional expression of mechanosensitive Piezo1 channels in the aggressive human melanoma SK-MEL-2 cell line. We found that chemical activation of Piezo1 by its agonist, Yoda1, prevents melanoma spheroid formation; thus, Piezo1 could be a potential target for selective modulation aimed at the prevention of melanoma development.

## 1. Introduction

Melanoma is a highly aggressive type of skin cancer produced through the malignant transformation of melanocytes, and it is usually associated with a poor prognosis. Clinically, melanoma has several stages associated with migration and invasion of the cells through the skin’s layers, the rapid spreading of cells and the formation of tumors in multiple organs. Today, the active development of novel targeted drugs is underway, but the main problem is the emergence of resistance in melanoma to the applied methods of treatment [1,2]. Therefore, it is of primary importance to find more crucial signaling pathways that control the progression of this type of cancer. Mechanosensitive ion channels and molecules, especially those that mediate Ca^2+^ influx into the cell, regulate cancer development, metabolism and progression [3,4]. Recently, it was shown that the mechanosensitive TRPV2 channel regulates melanoma invasiveness and metastatic potential [3]. The high expression of Piezo1, another important mechanosensitive Ca^2+^-permeable ion channel, was detected in melanoma [3], and was shown to correlate with its malignant progression [4]. The main life-threatening stages of melanoma are metastasis formation and metastatic spread, which are mainly determined by cell adhesion regulators. Three-dimensional spheroids are better suited to the study of metastatic spreading due to the imitation in vivo conditions that can be observed during melanoma progression [5]. Here, we evidenced functional activity of Piezo1 channels in the aggressive human melanoma SK-MEL-2 cell line. We showed that the chemical activation of Piezo1 by its selective agonist, Yoda1, completely prevents melanoma spheroid formation, indicating a regulatory role of Piezo1 in this process.

## 2. Results and Discussion

**Figure 1:**
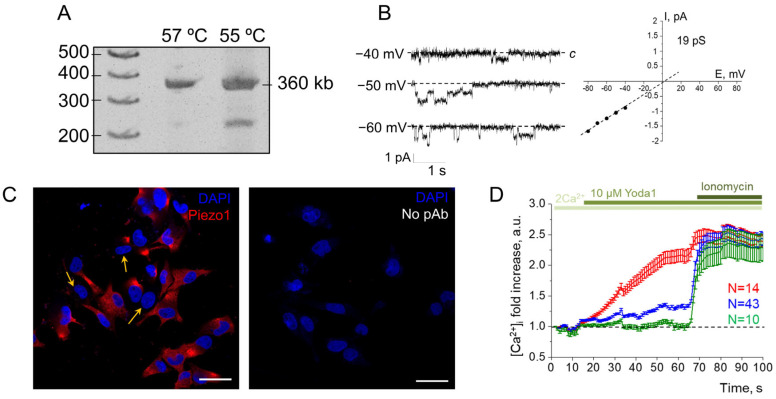
Functional expression of mechanosensitive Piezo1 channels in SK-MEL-2 cells. (**A**) RT-PCR showing the presence of hPIEZO1 mRNA in SK-MEL-2 cell lysates. The obtained length of the product (360 bp) was as expected. Original gel is available in Supplementary Figure S1. (**B**) Representative whole-cell recordings of Yoda1-induced Piezo1 activity in response to 1 µM Yoda1. Single-channel conductance and reversal potential were similar to Yoda1-induced Piezo1 channels described earlier [6]. c—closed state (zero current, baseline). (**C**) Immunofluorescence staining of Piezo1 in SK-MEL-2 cells. Red channel—Piezo1, blue channel—DAPI. Note that the number of cells is Piezo1-negative (marked by the arrows). Scale bar is 50 μm. As a control, staining of the cells only with fluorescent secondary antibodies was carried out (no pAb, no red fluorescence signal was observed). (**D**) Ca^2+^ responses of SK-MEL-2 cells to Yoda1 application in the representative experiment with n = 67 cells (from N = 3 independent Ca^2+^ measurements). Note that three populations of the cells could be distinguished (see Results and Discussion). Shown are mean fold changes (±S.D.) of Fluo8 fluorescence, normalized to the fluorescence at the starting point (first frame). Note that all cell populations had similar reactions to calcium ionophore ionomycin (5 μM), indicating the specificity of Yoda1-induced Ca^2+^ responses.

Firstly, we performed RT-PCR to confirm the presence of hPIEZO1 mRNA in the aggressive melanoma SK-MEL-2 cell line (Figure 1A). Further, to record the functional activity of endogenous Piezo1 channels in the plasma membrane, we used the patch-clamp technique. Previously, we have shown that 1 µM Yoda1, a selective agonist of Piezo1, could evoke single Piezo1-mediated channel activity in whole-cell experiments [6]. Consistent with this, we recorded single-channel Piezo1 currents in melanoma cells in response to the application of 1 µM Yoda1. The single-channel properties of Yoda1-induced Piezo1 channels (Figure 1B) were similar to those reported earlier [6]. The channels were activated in seven out of nine experiments, indicating that several cells seem to have no functionally active Piezo1 in their plasma membranes. Consistently, immunofluorescent staining revealed that about 25% (96 out of 261 cells) of SK-MEL-2 cells were Piezo1-negative (cells were counted from nine images from N = 3 independent staining experiments, Figure 1C). As Piezo1 channels provide physiologically relevant pathways for Ca^2+^ entry in various cells and tissues, Ca^2+^ measurements were carried out for additional verification of the obtained results (Figure 1D). We found that Yoda1 application resulted in a [Ca^2+^]_i_ increase, further confirming the presence of functional Piezo1 channels in SK-MEL-2 cells. Interestingly, Ca^2+^ imaging allowed us to distinguish three types of cell responses: (1) cells with significant increases in [Ca^2+^]_i_ (about 2-fold, red line), (2) cells with slow moderate increases (about 1.3 fold, observed in the majority of the cell population, blue line), and (3) cells with no Ca^2+^ entry in response to Yoda1 (green line). These results confirm the variations in Piezo1 expression in SK-MEL-2 cells observed in immunofluorescent staining and patch-clamp experiments. Taken together, our data show that SK-MEL-2 cells have functionally active Piezo1 channels in their plasma membranes, whose activity could be evoked by Yoda1, a selective chemical Piezo1 activator. An intriguing result is the presence of a number of the cells in which no Piezo1 staining or activity was detected. This phenomenon could be explained by various factors that could be revealed and analyzed in further studies.

**Figure 2:**
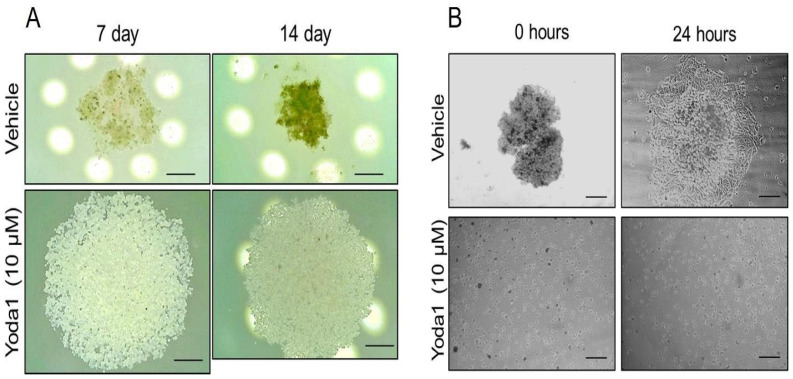
Yoda1-induced Piezo1 activation prevents spheroid formation from malignant melanoma SK-MEL-2 cells. (**A**) Shown are representative microphotographs of SK-MEL-2 spheroids in control (vehicle, 0.1% DMSO) and in the presence of 10 μM Yoda1 on 7th and 14th days after hanging drop initiation from N = 5 independent spheroid formation assays (total number of spheroids = 140). Note the more compact size of melanoma spheroids under control conditions (with further compaction from 7th and 14th days) compared to Yoda1-treated cells. Scale bar is 300 μm. White circles are the light source (light-emitting diodes) of the upright microscope. (**B**) Representative images of the melanoma spheroids at 0 h and 24 h after their collection and plating on the Petri dishes. Note that no spheroids were formed in the presence of Yoda1 (cell suspension was observed). After 24 h, the melanoma cells migrated from the control spheroids, indicating their viable status. At the same time, the cells collected from Yoda1-containing hanging drops remained round (were not able to spread) and were non-viable (were positively stained with propidium iodide, data not shown). Scale bar is 300 μm.

Cell aggregation in 3D spheroids is an important step in melanoma progression where cells primarily have to change their microenvironment and mechanical properties, as well as their morphology, polarization and cell–cell contacts. These steps could be critically controlled via the functioning of various ion transporters, including mechanosensitive channels. Thus, we investigated whether the selective chemical activation of Piezo1 could have any effect on melanoma spheroids by adding 10 µM Yoda1 to SK-MEL-2 suspension during the formation of spheroids. Importantly, we detected the perturbation of spheroid formation in the presence of Yoda1: firstly, no compaction of the cells was observed (Figure 2A), whereas the control cells became compacted after 7 days from the start of spheroid formation, and the compaction continued (to the 14th day). Further (after 14 days), the control- and Yoda1-containing drops were transferred to Petri dishes to confirm stable spheroid formation. Evidently, no spheroids were formed in the presence of Yoda1, whereas the control cells formed distinct compact spheroids (Figure 2B). After 24 h on the adherent surface, the cells from the control spheroids had spread out, whereas the Yoda1-treated cells remained round, were unable to spread and were non-viable (Figure 2B). Thus, the addition of Yoda1 completely blocked spheroid formation from aggressive human melanoma SK-MEL-2 cells.

The first key step in spheroid formation is the transfer of the cells to suspension, which significantly affects their shape and plasma membrane curvature compared to adherent cells. As membrane curvature was shown to strongly affect Piezo1 distribution [7], as well as the activation potential of Yoda1 [8], we analyzed the action of Yoda1 on the SK-MEL-2 cells in the suspension. We hypothesized that Yoda1 could have a strong effect on the suspension of melanoma cells, and this could potentially underlie the prevention of spheroid formation. Thus, we tested whether the addition of Yoda1 to the cell suspension could affect their spreading and viability (Supplementary Figure S2). Our assays showed only a small (or non-significant) effect of 30 µM Yoda1 (note the higher concentration used compared to spheroid formation) on SK-MEL-2 cells. Particularly, the number of spread cells in the presence of Yoda1 was slightly lower at the early stage (after 20 min); however, no significant changes were detected at later time points (Supplementary Figure S2A), which also confirms the viability of the cells. Also, Yoda1 had no effect on cell shape, but caused a slight decrease in cell size (Supplementary Figure S2B). Thus, we did not detect any short-term effects of selective Piezo1 activation by Yoda1 on SK-MEL-2 cell viability. Furthermore, Zhang et al. demonstrated that Yoda1 promoted the malignant behavior of melanoma [6]; thus, we could have expected the enhancement of spheroid formation (rather than its perturbation) by Yoda1 in our study. However, the complete inhibition of spheroid formation from SK-MEL-2 melanoma cells caused by selective Piezo1 activation may reflect the significant differences in Piezo1-dependent signaling pathways between 2D and 3D cell cultures.

The lack of a significant effect of Yoda1 on melanoma cell suspension could indicate that the perturbation of melanoma spheroid formation in the presence of Yoda1 occurs at later stages of the process. It should be noted that no specific cell–cell interactions occur between the cells in suspension (at least during a short timescale) or during cell spreading, where cell–surface interactions prevail. Thus, we speculate that Piezo1 activation probably affects the cell–cell interactions that were observed in the 3D culture of SK-MEL-2 cells. It is of specific interest to analyze the mechanisms that underlie the inhibition of melanoma spheroid formation through the selective activation of Piezo1 channels. Three-dimensional structure formation occurs through the sequential establishment of cell–cell contacts, in which adhesion molecules such as integrins and cadherins are the key players [9]. Interestingly, both cadherins and integrins were shown to interact with or be regulated by Piezo1 and v.v. [10,11]. Thus, the study of the possible regulation of integrin/cadherin activity and interactions by Piezo1 during spheroid formation could be a promising experimental goal of future research on the prevention of tumor formation. 

## 3. Materials and Methods

### 3.1. Cells and Reagents 

SK-MEL-2 human malignant melanoma cell line (ATCC^®^ HTB-68™, RRID: CVCL_0069) was purchased from Biolot, St. Petersburg, Russia. Cells were cultured in RPMI 1640 medium (Biolot, Russia) containing 10% fetal bovine serum (Biowest, Rui de Caille, France) and antibiotic gentamicin (80 µg/mL, Biolot, Russia) in humidified incubator at 37 °C and 5% CO_2_. For patch-clamp studies, fluorescent staining and Ca^2+^ imaging, the cells were plated on glass coverslips 2–3 days before the experiments. Selective chemical Piezo1 activator 2-[(2,6-dichlorophenyl) methylsulfanyl]-5-pyrazin-2-yl-1,3,4-thiadiazole (Yoda1) was purchased from Tocris Bioscience (Ablington, United Kingdom, cat no 5586). Ca^2+^ ionophore ionomycin was purchased from Thermo Fischer Scientific (Waltham, MA, USA).

### 3.2. RT-PCR 

Total RNA and cDNA were obtained as described in previous studies [6,7]. The primer sequences for hPIEZO1 were 3′-CCAGAACAGGTATCGGAAG-5′ (reverse) and 5′-TGCTGTACCAGTACCTGCTG-3′ (forward). The cycling conditions were as in previous studies [8]. The number of PCR cycles was 30, and the optimal annealing temperature for hPIEZO1 primers is 57 °C.

### 3.3. Electrophysiology

A detailed description of the whole-cell mode of the patch-clamp technique used to record single ion channels in the plasma membrane is available in our publications [6,12]. Cytosol-like solution contained (in mM) 140 KAsp, 5 NaCl, 1 MgCl_2_, 2 EGTA, 20 HEPES/TrisOH and CaCl_2_ to establish intracellular Ca^2+^ concentration ([Ca^2+^]_i_) at 10 nM. The extracellular chamber solution contained 145 NaCl, 2 CaCl_2_, 1 MgCl_2_ and 10 HEPES/TrisOH. pH of all solutions was set at 7.3. Yoda1 was applied to the extracellular solution.

### 3.4. Immunofluorescence

Polyclonal primary antibodies against extracellular domain of Piezo1 were used (KD/KO validated, from Proteintech, Manchester, United Kingdom, Cat No 15939-1-AP, RRID: AB_2231460). Cells were fixed with 4% paraformaldehyde (15 min at room temperature, RT), blocked with 10% goat serum (1 h, RT) and incubated with 1:100 Anti-Piezo1 antibodies (overnight, at 4 °C). Then, the cells were incubated with 1:200 secondary goat anti-rabbit-Cy3 fluorescent antibodies (Santa Cruz, TX, USA, for 1 h, RT). Cell nuclei were counterstained with DAPI (0.05 μg/mL, 30 min, RT). Cells were mounted on glass slides using Vectashield mounting media (Vector Laboratories, Newark, CA, USA) and visualized using an Olympus FV3000 (Olympus Corporation, Shinjuku, Tokyo, Japan) confocal microscope.

### 3.5. Calcium Imaging

Ca^2+^ measurements were performed as described earlier [13]. Briefly, the cells were loaded with 5 μM Fluo8-AM fluorescent Ca^2+^ probe (AAT Bioquest, Pleasanton, CA, USA) for 45 min in a Ca^2+^-containing solution (at 37 °C and 5% CO_2_, in the dark); then, the cells were incubated for 15 min without the dye. The Ca^2+^-containing solution consisted of (in mM) 150 NaCl, 4.5 KCl, 2 CaCl_2_, 1 MgCl_2_ and 10 HEPES/TrisOH. At the start of the experiment, cells were placed in Ca^2+^-containing solution, and basal fluorescence signals were recorded for 15 s. After that, 10 µM Yoda1 was applied to the cells. The experiment was performed at least 3 times.

### 3.6. Spheroid Formation Assay 

The melanoma spheroids were formed using a hanging drop method. Five independent experiments with a total number of 140 hanging drops for each experimental condition (control and Yoda1-treated cells) were conducted. Firstly, SK-MEL-2 cells were trypsinized, collected in culture medium and counted using a Countess II Automated Cell Counter (Thermo Fisher Scientific, Waltham, MA, USA). The cell concentration in suspension was adjusted to 2 × 10^5^ cells/mL (equal to 7.000 of cells in 35 µL drop). The total cell volume was divided into two, and 10 µM Yoda1 or an equal amount of DMSO (vehicle, 0.1%) was added. The drops were deposited onto the cover of a Petri dish, the drops were overlaid, transferred to a CO_2_ incubator and monitored daily using an upright digital microscope. After 14 days of formation, the drops were collected and plated on Petri dishes to obtain microphotographs of the spheroids.

### 3.7. Cell Spreading and Morphology Assays

Cells were trypsinized and resuspended in full medium, and vehicle (0.3% DMSO) or 30 µM Yoda1 was added to the cells. After that, cell suspensions were plated on Petri dishes. At least five fields of view for control- or Yoda1-treated cells were captured every 20 min, and the number of spread/non-spread cells was counted. For morphology analysis, the contours of the cells were traced manually in ImageJ using the “Freehand selection” tool. Cell area and shape descriptors were measured using the “Analyze–Measure” commands. A total number 100–110 cells were analyzed for each experimental condition at each timepoint.

### 3.8. Statistics

The statistical analysis was performed in GraphPad Prism 8.0 (GraphPad Software, USA), *p* < 0.05 was considered significant. The statistical tests used for data comparison are indicated in the corresponding figure legends.

## Data Availability

Data supporting the reported results are available upon request.

## Supplementary Materials

The following supporting information can be downloaded at: https://www.mdpi.com/article/10.3390/ijms242115703/s1.
