# Supplementary material for: Piezo1 Activation Prevents Spheroid Formation by Malignant Melanoma SK-MEL-2 Cells
Source: Int J Mol Sci. 2023 Oct 28;24(21):15703. doi: 10.3390/ijms242115703 (PMC10648948; doi:10.3390/ijms242115703)
Supplement: Supplementary file 1 [file ijms-24-15703-s001.zip › Supplementary.pdf]

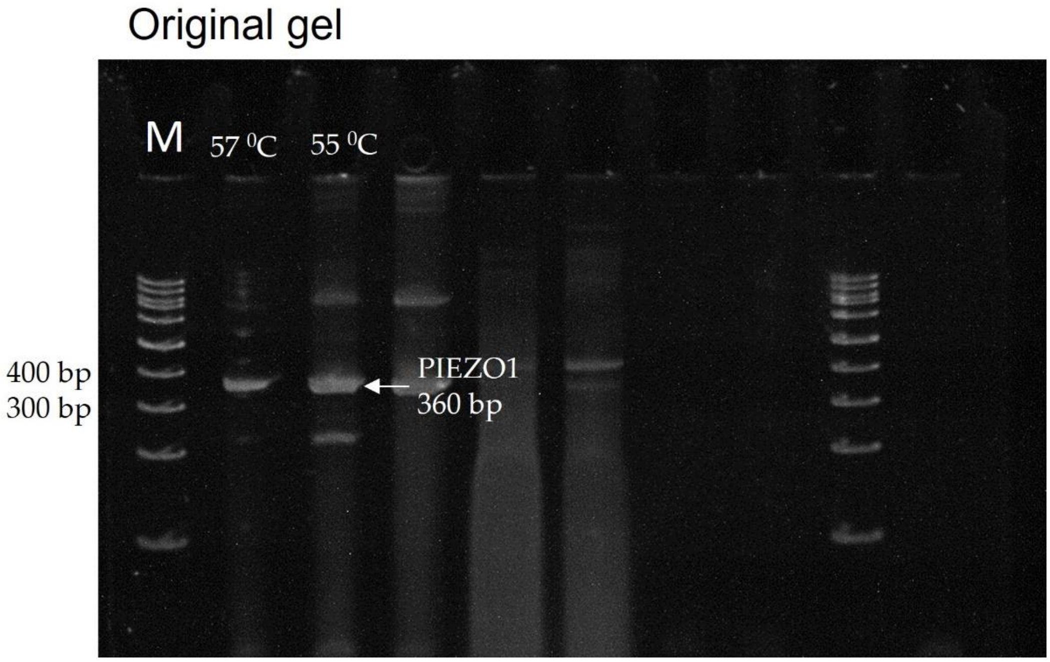

Supplementary Figure S1. Original gel showing the presence of hPIEZO1 mRNA in SK-MEL-2 cell lysates.

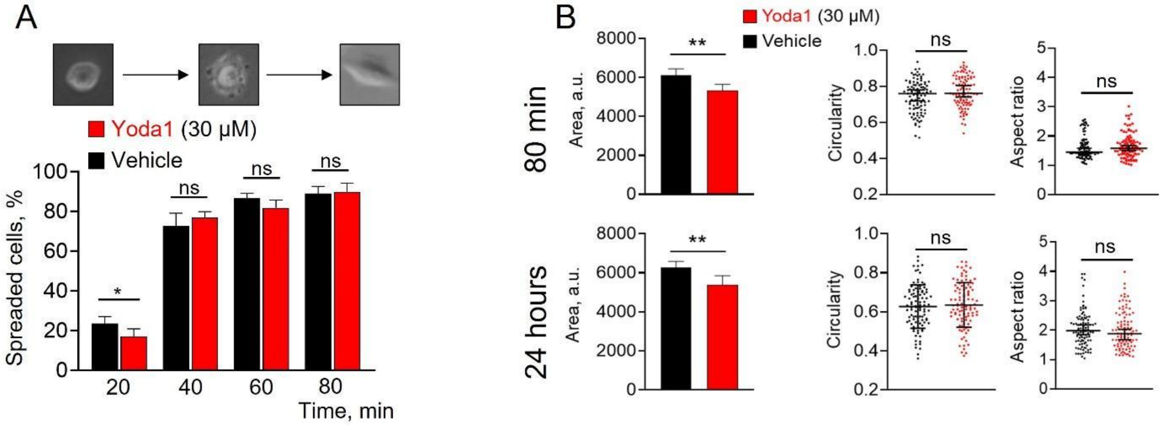

Supplementary Figure S2. The effect of Yoda1 on SK-MEL-2 spreading and morphology. (**A**): Microphotographs showing the subsequent steps of SK-MEL-2 spreading. Round cells were considered as non-spreaded (left box), cells at center and right boxes were considered as spreaded. Shown are percentages of spreaded cells (± S.D.) at each timepoint. A slightly lower (significantly different, p=0.0236, Student's t-test) percent of spreaded cells in the presence of Yoda1 after 20 min from the start of the experiment whereas no significant differences between experimental conditions were detected at later time points (40, 60 and 80 min). Successful spreading of the cells indicates their viability. (**B**): The effect of Yoda1 on SK-MEL-2 morphology. The cell area, circularity and aspect ratio of melanoma cells after 80 min and 24 hours from the beginning of the experiment were calculated. A small, but significant (**p<0.01, Mann-Whitney test) decrease (by 15-17%, compared to control) of cell area in the presence of Yoda1, whereas cell circularity and aspect ratio were not significantly different (Student's t-test and Mann-Whitney test, respectively). Shown are medians and 95% confidence intervals for cell area and aspect ratio, and mean ± S.D. for cell circularity. ns - the differences are non-significant.
