# Supplementary figures and images for: Piezo1 Activation Prevents Spheroid Formation by Malignant Melanoma SK-MEL-2 Cells
Source: Int J Mol Sci. 2023 Oct 28;24(21):15703. doi: 10.3390/ijms242115703 (PMC10648948; doi:10.3390/ijms242115703)

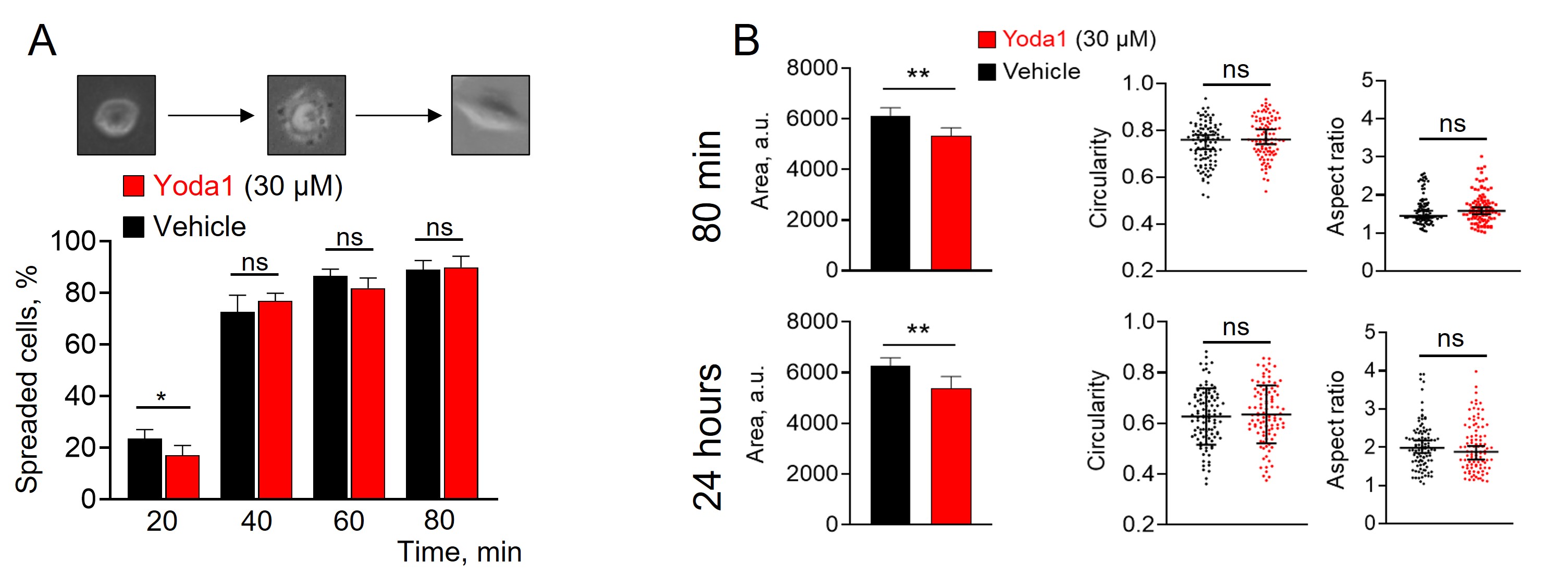

Supplement: Supplementary file 1 [file ijms-24-15703-s001.zip › Suppl FigS2.jpg]
